# Exergaming Platform for Older Adults Residing in Long-Term Care Homes: User-Centered Design, Development, and Usability Study

**DOI:** 10.2196/22370

**Published:** 2021-03-09

**Authors:** Charlene H Chu, Renée K Biss, Lara Cooper, Amanda My Linh Quan, Henrique Matulis

**Affiliations:** 1 Lawrence S. Bloomberg Faculty of Nursing University of Toronto Toronto, ON Canada; 2 Institute for Life Course and Aging University of Toronto Toronto, ON Canada; 3 KITE-Toronto Rehabilitation Institute University Health Network Toronto, ON Canada; 4 Department of Psychology University of Windsor Windsor, ON Canada; 5 Dalla Lana School of Public Health University of Toronto Toronto, ON Canada; 6 Department of Mechanical & Industrial Engineering University of Toronto Toronto, ON Canada

**Keywords:** user-centered design, aged, long-term care, nursing homes

## Abstract

**Background:**

Older adults (OAs) residing in long-term care (LTC) homes are often unable to engage in adequate amounts of physical activity because of multiple comorbidities, including frailty and severe cognitive impairments. This level of physical inactivity is associated with declines in cognitive and functional abilities and can be further compounded by social isolation. Exergaming, defined as a combination of exercise and gaming, has the potential to engage OAs in exercise and encourage social interaction. However, previously used systems such as the Nintendo Wii are no longer commercially available, and the physical design of other exergames is not suitable for OAs (ie, fall risks, accessibility issues, and games geared toward a younger population) with diverse physical and cognitive impairments.

**Objective:**

This study aims to design and develop a novel, user-centered, evidence-based exergaming system for use among OAs in LTC homes. In addition, we aim to identify facilitators and barriers to the implementation of our exergaming intervention, the MouvMat, into LTC homes according to staff input.

**Methods:**

This study used a user-centered design (UCD) process that consisted of 4 rounds of usability testing. The exergame was developed and finalized based on existing evidence, end user and stakeholder input, and user testing. Semistructured interviews and standardized and validated scales were used iteratively to evaluate the acceptability, usability, and physical activity enjoyment of the MouvMat.

**Results:**

A total of 28 participants, 13 LTC residents, and 15 staff and family members participated in the UCD process for over 18 months to design and develop the novel exergaming intervention, the MouvMat. The iterative use of validated scales (System Usability Scale, 8-item Physical Activity Enjoyment Scale, and modified Treatment Evaluation Inventory) indicated an upward trend in the acceptability, usability, and enjoyment scores of MouvMat over 4 rounds of usability testing, suggesting that identified areas for refinement and improvement were appropriately addressed by the team. A qualitative analysis of semistructured interview data found that residents enjoyed engaging with the prototype and appreciated the opportunity to increase their PA. In addition, staff and stakeholders were drawn to MouvMat’s ability to increase residents’ autonomous PA. The intended and perceived benefits of MouvMat use, that is, improved physical and cognitive health, were the most common facilitators of its use identified by study participants.

**Conclusions:**

This study was successful in applying UCD to collaborate with LTC residents, despite the high number of physical and sensory impairments that this population experiences. By following a UCD process, an exergaming intervention that meets diverse requirements (ie, hardware design features and motivation) and considers environmental barriers and residents’ physical and cognitive needs was developed. The effectiveness of MouvMat in improving physical and cognitive abilities should be explored in future multisite randomized controlled trials.

## Introduction

### Background

Globally, 3%-5% of the 962 million older adults (OAs) [1] live in some form of long-term care (LTC) home to receive assistance with their activities of daily living and primary medical care [2]. There is significant growth in the demand for LTC in almost every country; for instance, Canada’s demand for LTC is twice that of its current capacity [3]. Routine physical activity (PA) is a widely recognized component of healthy aging among OAs [4-6]. However, it is difficult for OAs who reside in LTC homes (herein referred to as residents) to engage in adequate amounts of PA because of multiple comorbidities, including frailty and severe cognitive impairments [1,7]. The sedentary nature of living in LTC homes is well documented [8], and this level of physical inactivity significantly increases the likelihood of further declines in cognitive and functional abilities, especially in the first few months after OAs are admitted to LTC homes [9]. The high prevalence of physical and cognitive impairments among LTC residents also compounds social isolation [10,11], a risk factor that negatively influences cognitive [12] and mental health.

The World Health Organization [13] reports a critical need for interventions that can help OAs maintain their physical and cognitive health and reduce social isolation. Given the globally aging population and increased pressure on international LTC sectors, there is a need for effective, translatable, and sustainable interventions that can provide opportunities for physical, cognitive, and social stimulation to LTC residents. Exergaming products are a safe way to improve these outcomes in OAs [14-17]. The current literature suggests that exergames have positive social, cognitive, and physical effects [18-21]. Exergaming technology is described as interactive exercise-based games, whereby players are able to engage in physical and cognitive activities through a game. Exergaming provides an enjoyable means to motivate LTC residents and offers opportunities to use both motor and cognitive skills. The mechanism of this benefit is supported by the cognitive enrichment hypothesis [22], which states that the collective behaviors of an individual have a meaningful positive impact on cognition and function in old age. Thus, exergaming may exert its positive effects by providing simultaneous physical, cognitive, and social stimulation, which in turn mitigates functional decline.

However, an ongoing analysis of the existing body of evidence regarding the validity of exergaming as an activity for OAs has revealed that many studies are outdated [23] and are predominantly based on gaming systems that are no longer commercially available, such as the Nintendo Wii or the XBOX Kinect [24]. These popular systems were not designed for residents in institutional settings, such as LTC homes, and therefore have several features that make them less suitable for this population. First, interactive gaming platforms that encourage movement, such as Nintendo Wii Yoga and Dance Dance Revolution, use raised platforms or metal pads and mats posing a fall risk and accessibility issues for residents with physical disabilities or who require wheelchairs [19]. Although these gaming systems are accessible for research purposes, the physical design poses safety hazards for residents [25]. Second, the multiple small components, such as handheld controllers, are also difficult to sanitize, posing a risk to infection control. Third, the games offered are generally designed around the interests of younger populations and often encompass small text, fast flashing screens with visual distraction, and unrelatable game concepts that are more difficult for OAs to comprehend (eg, collecting imaginary characters) [26]. In addition to the gaming system limitations of previous work, from a methodological perspective, there are only a small number of exergaming studies that included residents with cognitive impairment [18], despite 87% of residents living with some type of cognitive impairment [27]. Furthermore, we were unable to find studies that aimed to co-create an interactive exergaming platform for LTC residents from ideation to validation and implementation in LTC homes. These methodological shortcomings not only point to a discrepancy between the state of the evidence and the advanced needs of residents and the LTC environment, but also underscore the urgent need for gaming systems that are applicable to this population in their congregate home settings.

In response, we used a design science research strategy to develop an exergaming platform that can promote the increased general PA, cognitive stimulation, and social engagement of residents in LTC homes. We applied a user-centered, iterative design process [28,29] to center the experiences and perspectives of residents and other stakeholders to address the safety, accessibility, and practical disadvantages of existing systems. The vision was to create an exergaming platform that was versatile and fully adaptable to meet the range of physical abilities of LTC residents (eg, able to encourage lower body activity in residents who are able to ambulate with or without a gait aid or engage the upper body of residents who are in wheelchairs or unable to ambulate) and meet the diverse social needs of residents in LTC (ie, ability to play single-player or multiplayer games). The platform software should be customizable with respect to difficulty levels, and the hardware should have the capability to be physically reconfigured to meet the needs of the participants.

### Objectives

The first aim of this paper is to describe the iterative process of developing a prototype of a novel exergaming system, the MouvMat, designed for residents with varying cognitive and physical abilities to improve its acceptability, usability, and enjoyment to residents and stakeholders, including staff and family members. The second aim is to identify the facilitators and barriers to the implementation of the MouvMat in LTC homes according to staff stakeholders.

## Methods

### Study Enrollment

Following internal alpha testing, our research team collaborated with 2 LTC homes located in Toronto, Ontario, Canada, to recruit the study participants. Various recruitment strategies included posters and direct enrollment by an LTC home staff collaborator. If available, participants were invited to participate again for each of the 4 rounds of usability testing. Study inclusion criteria for residents were as follows: participants should (1) be a resident of an LTC home; (2) be aged ≥65 years; (3) be able to read, speak, and write in English; and have a score above 10 on the Mini-Mental Status Examination (MMSE) [30]. Collaterals were eligible if they were (1) a family member, substitute decision maker (SDM), or personally hired caregiver of an OA living in an LTC facility; (2) able to read, speak, and write in English; and (3) aged ≥18 years. LTC home staff were eligible if they were able to communicate in English and were currently working in an LTC facility. Study eligibility was assessed as part of the informed consent form. Once enrolled, a research assistant collected demographic information and familiarity with electronic devices and game technologies, administered the MMSE [31], and completed the Frailty in Nursing Homes Scale (FRAIL-NH) [32], with the latter two measures administered to residents only. Frailty (ie, FRAIL-NH), cognitive status (ie, MMSE), and presence of sensory or motor impairments were collected to ensure and demonstrate that MouvMat was accessible to LTC residents with varying cognitive and physical abilities.

### User-Centered Design Process

A robust 3-phase user-centered design (UCD) process was applied [28,29]. The first phase of the exergaming platform design was based on developing an explicit understanding of residents and LTC home environments with residents and LTC staff. Interviews, a SWOT (strengths, weaknesses, opportunities, and threats) analysis, and paper mock-ups were completed in line with ISO 9241-210:2019 (human-centered design standards) [29] that have been described elsewhere [33]. Potential end users, including residents in LTC and LTC staff and health care providers, were involved in all steps of prototype conceptualization and development. The second phase included creating a testable prototype of the exergaming platform and gathering feedback from stakeholders and end users using a think-aloud method [34] to assess the acceptability of the idea and to inform the development of a high-fidelity prototype [35,36].

Finally, the third phase was focused on conducting 4 rounds of usability testing of the high-fidelity prototype with one or more games (game descriptions are provided in Multimedia Appendix 1) and conducting summative user feedback with each iteration of the exergame. Participants enrolled in the study (residents, family members or SDM, and staff) were invited to complete 1-hour user testing sessions that involved using the prototype with a concurrent think-aloud method. Each of the 4 user testing sessions followed the same sequence and involved (1) informed consent and collection of demographic and characteristic information, as well as cognitive and frailty measures (MMSE and FRAIL-NH); (2) engagement with the prototype using the talk-aloud method; (3) quantitative measures; and (4) a semistructured interview informed by an interview guide.

To start the user testing session, a safety checklist was first filled out by a team member to mitigate fall risk during testing. Each user testing session was supervised by at least one researcher and game designer or engineer who were also present to take notes on system functionality and usability. The checklist comprised 5 questions, each answered with either yes or no responses to (1) confirm whether the participant was wearing nonslip footwear, glasses, and hearing aids, if needed, and whether the participant was not feeling tired or drowsy and (2) ensure that the testing environment was distraction free and quiet before reading the instruction script. Distractions and background noise can make the instructions difficult to hear, which may cause confusion, so it was important to verify that the participant and the testing conditions were safe before proceeding with the testing. If any condition was not met, a research team member would contact the staff or the testing session would be rescheduled to another time.

During the testing, team members observed and documented the ergonomic navigation of participants and the emotional responses of participants while engaging with the exergame. Functionality and participant performance (ie, the number of games played, number of levels completed, and number of errors) were documented during each session. In addition, behavioral observations and participant comments from the think-aloud method during the use of the MouvMat were recorded [37]. Observations are a central activity in UCD because they provide insights into how OAs were physically navigating on and around the MouvMat and how they interacted with the technology, for example, their facial or verbal expressions when they heard audio sounds, saw blinking lights, or successfully completed a level. Each participant was closely observed by multiple team members to ensure that small cues were not missed and each team member documented their own observations, which were later discussed as a group. During interaction with the prototype and talk-aloud responses, researchers avoided providing feedback or extensive comments to participants so as to minimally affect their potential responses to subsequent measures. Participants who were nonweightbearing or immobile in a wheelchair were provided a handheld device to point and tap on the system, whereas those who were mobile were able to stand up and tap the system with their foot. LTC residents’ sessions were completed individually or with a family member, SDM or caregiver present if this was their preference or if required.

Each user testing session was concluded with a semistructured interview focused on 6 constructs (functionality, usability, accessibility, enjoyment, acceptability, and design) to gather feedback about the game, hardware, and interface and to enhance our understanding of those concepts (usability, acceptability, and enjoyment) that were also measured with standardized and validated tools. The interviews were audio recorded, transcribed, and analyzed using thematic analysis [38] after each round. The research team would discuss the participants’ feedback, themes, and redesign the prototype to make appropriate changes that would better meet user needs in subsequent rounds of usability testing. If there were behaviors noted during the observation, the participant was asked to clarify or explain during the interview. For example, if the resident laughed when playing the game, they would be asked what specifically triggered their laughter, or if they asked a question about the game while playing, we inquired about the specific aspect they found confusing. Each participant received an honorarium for the time following the completion of the study. This iterative process was completed in 4 separate iterative rounds between July 2018 and December 2019 (approximately 18 months). The University of Toronto Research Ethics Board (RIS#37453) approved the study.

### Outcome Measures

In total, 3 main standardized and validated scales were used to evaluate the acceptability, usability, and PA enjoyment of the MouvMat. Acceptability refers to the values, judgments, and beliefs about the effectiveness, risks and benefits, and perceived usability of a treatment or innovation and is a primary determinant of end user uptake [39]. An adapted modified Treatment Evaluation Inventory (m-TEI) [40,41] was used to assess the acceptability of residents and stakeholders (ie, collaterals and staff). Stakeholders play a critical role in LTC, and their perceptions can either positively or negatively influence residents’ engagement with interventions [39]. Next, the System Usability Scale (SUS) [42,43] was used to evaluate the subjective usability of newly developed devices and systems. The SUS is a widely used validated and reliable scale consisting of 10 items and a score ranging from 0 to 100, with 68 representing the minimally acceptable usability score. Third, the 8-item Physical Activity Enjoyment Scale (PACES-8) [44] is a brief measure of subjective enjoyment of physical exercise validated in OAs [44]. Enjoyment is a relevant measure because it is both an important predictor and outcome of PA participation in OAs [45]. Furthermore, OAs’ anticipated enjoyment from physical activities can predict their adoption of and motivation to engage in physical activities [46,47].

### Data Analysis

Participant demographics were summarized using descriptive statistics. Questionnaire scores from m-TEI, SUS, and PACES-8 were compared across the four usability testing sessions and between participant groups (ie, residents, family members, and staff). We tested for differences across groups and testing sessions using linear mixed models fit by maximum likelihood, which allows for the analysis of repeated, longitudinal assessments in which there are cases of missing data [48]. Analyses were run in the GAMLj module of Jamovi software [49], and the model for each outcome measure included a random intercept for participants, with fixed effects for group (residents, family members, and staff or administrators) and usability testing sessions.

A qualitative content analysis approach [50] of the transcribed semistructured interviews was undertaken to deepen our understanding of the quantitative measures, identify facilitators and barriers, and gather information to inform technology development. Immediately after each session, the researchers and engineer or game designer (CC, RB, and HM) discussed their observational logs and field notes to compare observations, and these impressions formed the key themes and areas for refinement to improve the system. In addition, the team would debrief and discuss observations at the end of each testing day by applying an adapted constant comparison approach [51] with themes from the preceding sessions. The transcripts were sorted into emerging categorical themes related to improvements, facilitators, and barriers. This process was facilitated by 2 researchers using NVivo (NVivo Qualitative Data Analysis Software; QSR International Pty Ltd, version 11, 2016). In addition, member checking [52] was performed to confirm researchers’ interpretations of the participants’ statements in subsequent user testing sessions.

## Results

### Study Participants

Table 1 provides the demographic information of residents (13/28, 46%), staff (14/28, 50%), and family members (1/28, 4%), such as age, gender, measures of function, frailty status, and knowledge of technology. Residents had multiple physical, sensory, and cognitive impairments. All residents had vision issues, nearly half (6/13, 46%) had hearing issues, 38% (5/13) were prefrail who required moderate amounts of assistance in everyday function and PA, and 50% (6/12; data were missing for one participant) showed evidence of cognitive impairment when using an MMSE cutoff score of less than 26 [53]. The stakeholders included staff members comprised of personal support workers (8/15, 53%), managers (2/15, 13%), physiotherapist and physiotherapy assistant (2/15, 13%), life enhancement and activation therapists (2/15, 13%), and one family member who was the wife of a resident in the study (1/15, 7%). Staff were employed for at least 1 month to 4 years at their respective homes. Overall, 54% (7/13) to 77% (10/13) of residents had low to basic levels of knowledge of computers and handheld devices, respectively, whereas most staff had moderate knowledge.

**Table 1:** Participant demographics and characteristics (N=28).

Characteristics		Residents (n=13)	Staff members and family (n=15)^a^
Age (years), mean (range)		83.23 (66-97)	38.38 (19-70)
**Sex, n (%)**			
	Female	8 (61.54)	14 (93.33)
	Male	5 (38.46)	1 (6.67)
Years of education, mean (SD)		14.62 (2.59)	16.13 (2.47)
**Vision impairment^b^, n (%)**			
	Yes	13 (100)	8 (53.33)
	No	0 (0)	7 (46.67)
**Hearing impairment^c^, n (%)**			
	Yes	6 (46.15)	1 (6.67)
	No	7 (53.85)	14 (93.33)
**Mobility impairment^d^, n (%)**			
	Yes	11 (84.62)	0 (0)
	No	2 (15.38)	15 (100)
**Fear of falling^e^, n (%)**			
	Yes	3 (23.08)	N/A^f^
	No	10 (76.92)	N/A
**Gait aid use, n (%)**			
	No gait aid	2 (15.38)	N/A
	Walker	6 (46.15)	N/A
	Wheelchair	5 (38.46)	N/A
**Knowledge of computers, n (%)**			
	Basic	8 (61.54)	3 (21.43)
	Moderate	4 (30.77)	9 (64.29)
	Advanced	1 (7.69)	2 (14.29)
**Knowledge of handheld devices, n (%)**			
	Basic	11 (84.62)	3 (23.08)
	Moderate	1 (7.69)	8 (61.54)
	Advanced	1 (7.69)	2 (15.38)
**Mini-Mental Status Exam score, mean (SD)**		24.46 (5.30)	N/A
	>24, n (%)	7 (53.85)	N/A
	18-24, n (%)	4 (30.77)	N/A
	<18, n (%)	2 (15.38)	N/A
**Frailty in Nursing Homes Scale, mean (SD)**		3.6 (2.4)	N/A
	Nonfrail, n (%)	8 (61.54)	N/A
	Prefrail, n (%)	5 (38.46)	N/A

^a^Missing staff data for knowledge of PCs and knowledge of handheld devices questions; denominators for residents and staff and family members were 13 and 14, respectively.

^b^Visual impairments included self-reported vision problems, the use of glasses (eg, reading glasses), cataracts, and glaucoma.

^c^Hearing impairments included self-reported hearing problems and the use of unilateral or bilateral hearing aids.

^d^Mobility impairments included hemiparesis, unsteady gait, and uneven gait due to pain.

^e^Self-reported.

^f^N/A: not applicable.

### Usability Testing

**Figure 1:**
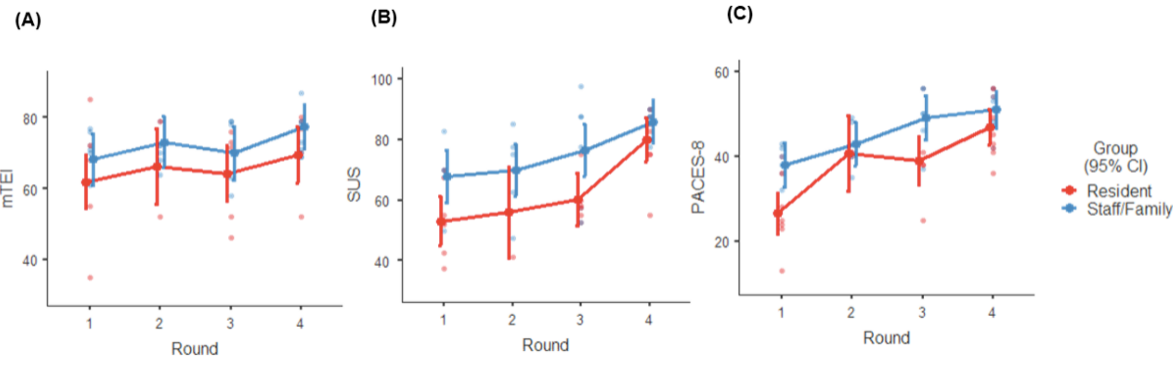
Quantitative measures of (A) acceptability, (B) usability, and (C) physical activity enjoyment among participants across 4 rounds of testing (means and CIs). m-TEI: Modified Treatment Evaluation Inventory; PACES-8: 8-item Physical Activity Enjoyment Scale; SUS: System Usability Scale.

In total, 4 iterative rounds were conducted to develop the MouvMat, and a summary of the development is presented in Tables 2 and 3. One resident participant refused data collection after enrollment; therefore, the analysis included 12 residents. Overall, 25% (4/12) of LTC residents provided responses for one round of usability testing, 50% (6/12) completed 2 rounds of testing, and 25% (4/12) attended 3 rounds of testing. Data from staff and family members were analyzed together. Among this group, the majority (11/15, 73%) attended 2 rounds of testing, with 20% (3/15) attending one round and 7% (1/15) attending 3 rounds. Not all individuals participated in all rounds of testing for reasons related to doctor or dentist appointments, day trips, and availability of the residents. The areas of refinement were primarily guided by observations during participant interactions with the system. After each round of usability testing, the SUS, PACES-8, and m-TEI were collected and are reported in Figure 1 (Multimedia Appendix 2 provides scores). The data from all rounds of usability testing were summarized and categorized according to the study objectives: acceptability, usability, and PA enjoyment.

**Table 2:** Summary of iterative development process presented in rounds 1 and 2.

Round	Description of prototype	Primary areas for refinement
1	Presentation: participants were presented with a low-fidelity prototype of the MouvMat, which consisted of 4 modular gaming squares. Each square was made of polyethylene terephthalate plastic with 59 RGB^a^ LEDs embedded and distributed in 6 parallel rows of 7 to 11 LEDs each, with no alignment of columns. Each square contained its own pressure sensor and was wired to an Arduino. Thus, there were multiple wires connecting each square to the Arduino Each side of the square measured approximately 28 cm (11 inches). A nonslip backing was on each square Programming: there were no games programmed into the interface because this first stage was to identify whether this type of platform would be tolerated by the residents. The lights were manually activated by a team member to emulate game play to see how residents interacted with this gaming format Audio: none Pressure sensors: none Peripheral accessory: wheel-bound participants were given a wooden stick with a circular platform at the end to use as the accessory 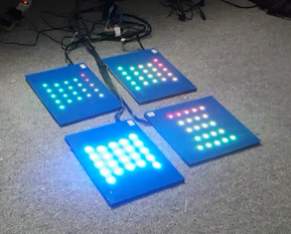 Image of prototype used in round 1	Participants did not like the multiple wires (because of fall risk) Requested to hear sounds for audio feedback Participants found it difficult to envision a game and wanted to experience *playing a game* The wooden stick provided to residents to emulate the peripheral device was reported by residents to be *a little heavy* and needed a larger surface area at the bottom to activate the squares
2	Presentation: 4 acrylic (polymethyl methacrylate) squares that were all centrally connected to the Arduino. There were 6 to 10 parallel rows of 7 to 11 LEDs in each row. Each side of the square measured approximately 28 cm (11 inches). A nonslip material was on the back of each square Programming: there was one game programmed similar to *Simon Says* programmed on the Arduino Audio: basic audio chimes played over a mid–low-fidelity speaker when the squares were activated. There were also short music clips that would play upon the successful completion of a sequence Pressure sensors: Velostat sheet (polymeric foil) Peripheral accessory: wheelchair-bound participants were given a plastic stick with a circular platform on a swiveling head to use as the accessory 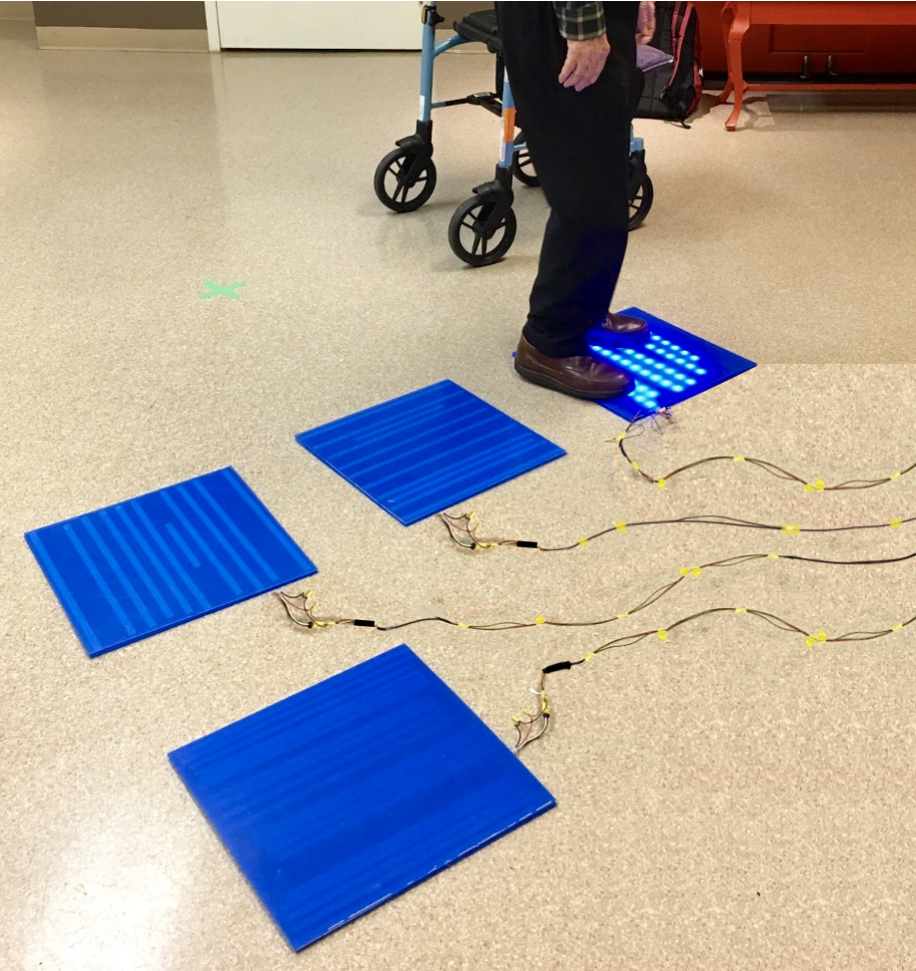 Image of prototype used in round 2	Participants suggested more squares for a bigger play space to experience more gaming options Some shoes were extending over the border of the square, so we needed larger squares to accommodate size 12 shoes Residents wanted to see more games The accessory’s circular platform was *too heavy* and not long enough according to residents The basic audio chimes that were added were not motivating enough The game was not responsive enough—the squares had to be stepped on multiple times to activate Staff identified that lifting the stack of squares would be *a bit too heavy* if they had to carry the squares from one recreation room to another room or unit From a development perspective, the programming of the games was time consuming, and we found the single Arduino was fragile and hard to upgrade

^a^RGB: red, green, blue.

**Table 3:** Summary of iterative development process presented in rounds 3 and 4.

Round	Description of prototype	Primary areas for refinement
3	Presentation: increased the number to 6 polycarbonate squares that were approximately 20% larger than the previous iteration (33 cm/13 inches side); each pad was reconfigured to have its own microcontroller that could be controlled wirelessly. The male-female connectors were replaced with magnets for quick assembly. Each square has an 8×8 matrix of RGB^a^ LEDs that can be controlled individually, providing the possibility of low-level graphics. To make each square lighter and easier to carry, we used a die-cutter to create custom antistatic high-density polystyrene cushioning to replace the plywood from the first 2 prototypes A master square is powered using a P10 connector, and the power is distributed to the other squares by the magnets Programming: there were 4 games—Simons Says, Memory Pairs, Scrabble, and Don’t Let the Music Stop. The squares work independently of each other and are connected to a Raspberry Pi server that controls the pads and provides an infrastructure for game development in high-level languages such as Python and JavaScript Audio: improved sound quality of chimes and audio voices and music played remotely Pressure sensors: Velostat sheet (Polymeric foil) Peripheral accessory: a telescopic aluminum stick to adjust the length and reach; features a circular foam platform on a swiveling head to use as the accessory 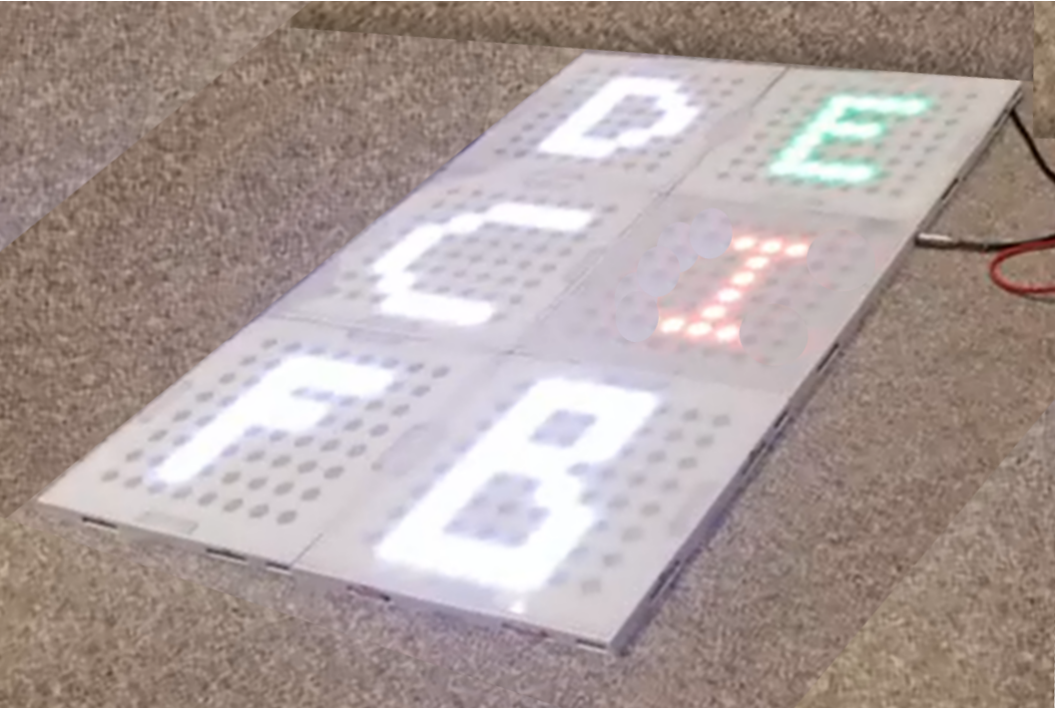 Image of prototype used in round 3	Participants suggested that the images on each square could be sharper or higher resolution Requested more games Requested more squares Requested music and improved sound quality Residents commented that the accessory could be more comfortable to hold in their hands
4	Presentation: the number of squares was increased to 9, each the same dimensions of iteration in round 3. A new 3D printed frame was created to accommodate the new stronger magnets that were partially embedded to prevent the separation of tiles. Each square has a 16×16 matrix of RGB LEDs to increase the resolution. A nonslip material was on the back of each square Programming: there were 5 games programmed in Python: Simons Says, Memory Pairs, Scrabble, Don’t Let the Music Stop, and Tic-Tac-Toe. New easy-to-use gaming interface that can be used on a tablet to select the game and adjust background music volume or mute it Audio: background soundtracks with volume adjustment, audio voices, better quality speakers Pressure sensors: Piezo sensor Peripheral accessory: adjustable telescopic pole with foam grip and foam circular bottom to optimize comfort, lightweight, and adjustability 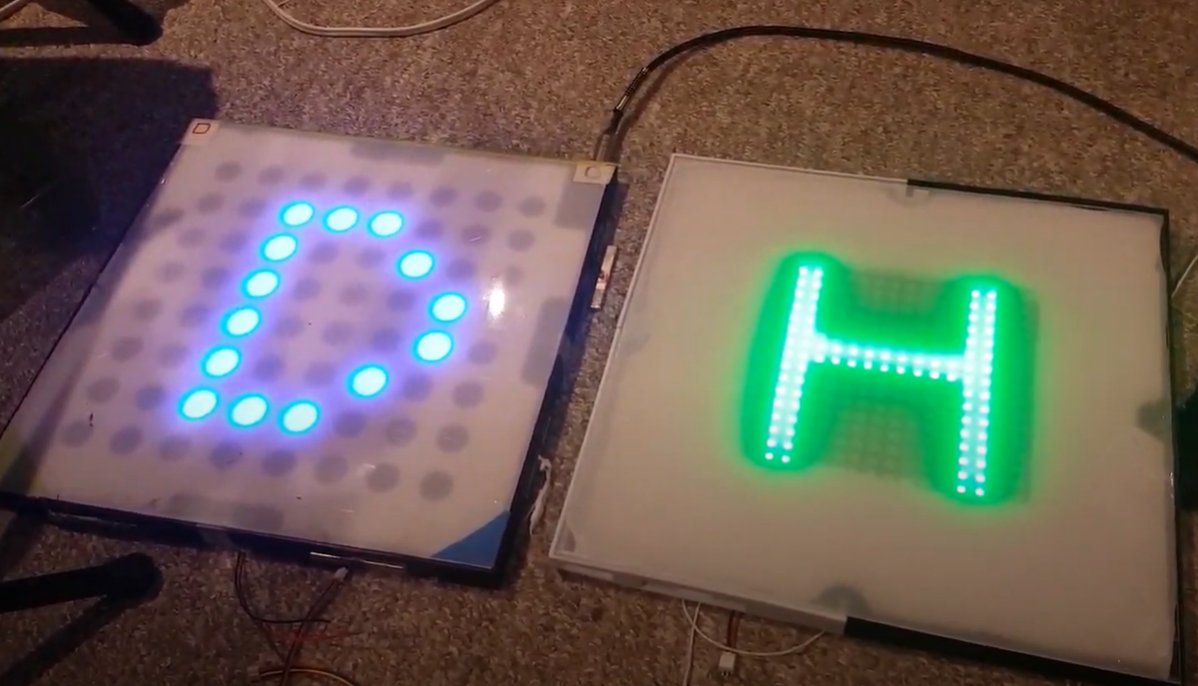 Image comparing a square from round 3 (left) with a square with a higher resolution display in round 4 (right) 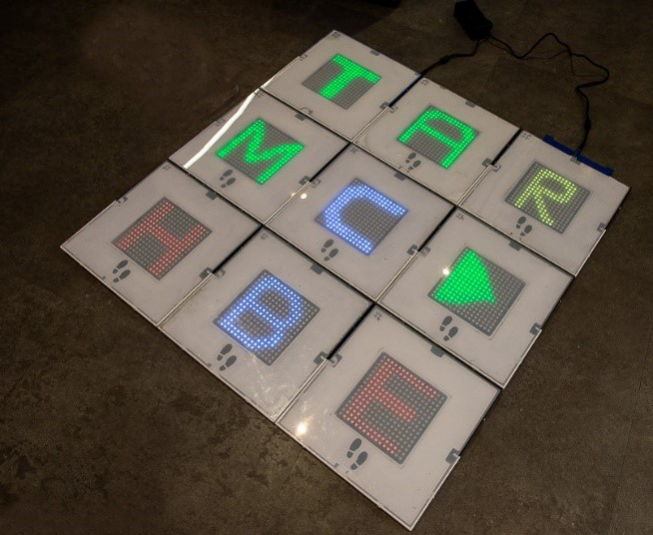 Image of a high-fidelity version used in round 4	Future development plans include but are not limited to the following: (1) adding ID readers for individual user identification and game selection; (2) replacing glued magnets with screws; (3) increasing the level of magnet embedding; (4) logging infrastructure; (5) including the possibility of playing the games on a regular computer, tablet, or cellphone over the web; (6) developing more games; (7) logging the infrastructure of performance data for automated user performance data collection; (8) adjusting the level of difficulty for each user or user group; and (9) increasing the resolution of presented graphics

^a^RGB: red, green, blue.

### Exergame Acceptability

Acceptability of end users and stakeholders is important to understand, as it impacts intervention use and uptake [39]. The results of the mixed linear model analysis of m-TEI scores (Figure 1) showed that, as modifications were made based on user feedback over the 4 rounds of testing, m-TEI scores increased, indicating improved acceptability (*F*_3,21.7_=3.92; *P*=.02). There was no significant effect of group (*F*_1,24.1_=2.59; *P*=.12) or interaction between group and round of usability testing (*F*_3,21.7_=0.07; *P*=.98).

This trend of increasing acceptability is consistent with participant responses during interactions with successive prototypes and comments during the semistructured interviews. During the initial testing session, residents and stakeholders all confirmed that they had not encountered an exergaming system or technology such as the MouvMat. On the basis of the items in the m-TEI, all respondents found the concept of the exergame—a set of interactive and configurable squares that the user engages with their feet or an accessory—to be acceptable. From round 1, the overall general reaction toward the MouvMat was scored *very positive* and scores continued to improve. Overall, based on common sense notions about regular activity, all respondents indicated on the m-TEI that the use of the MouvMat could improve PA, social activity, and cognitive stimulation. During the interviews, positive feedback from staff included:

I think it’s a really interesting idea. It is definitely something that would engage the residents in physical activity. This is something they can have in their rooms. It can be good for residents who prefer to be alone. I think that’s a really good idea.

P16, staff

Residents identified that the potential for PA in an upright or seated position was a strength that bolstered its acceptability. Residents were willing to use the MouvMat themselves, and staff members and family found it suitable for those who did not regularly exercise and particularly liked the use of a peripheral accessory for residents who are nonweightbearing or in wheelchairs.

Key determinants of acceptability that were identified in round 2 interviews included mitigating the potential risk of falls and ensuring that game use was intuitive rather than cognitively taxing. Falls or a loss of balance while standing or from moving square to square was the primary risk and harm mentioned by staff (5/15, 33%) and residents (4/13, 31%). To address this concern, the team highlighted the nonslip surface of the squares to participants during testing rounds and ensured that further development would address attributes that would reduce fall risk and fear of falling. A staff member said:

I don’t feel like I’m going to slip when I’m standing on it, but I think it would be easier for residents with rollators if the squares could be made more flush to the ground and larger.

Recreational therapist

To respond to this suggestion, the team also developed thinner squares that were closer to the ground made of high-density polystyrene in round 3. In addition, without any programmed games in the prototype in round 1, some residents (3/13, 23%) requested to play a programmed game to fully experience the cognitive and social aspects of game play:

I couldn’t figure out why the light went from one [square] to another or where to step first...I think playing an actual game with goals would help.

P02, resident

This sentiment revealed that games needed to be developed and that these future games needed features that are intuitive to users, such as a greeting message to inform the player of when the game was about to start. We responded to this resident request by programming games for subsequent rounds and integrating game design elements such as adding greeting messages and a count down with blinking lights in subsequent rounds. In the final round, we observed that participants appeared more eager to start playing the game as they were watching the countdown. The residents and stakeholders confirmed liking these intuitive game design aspects, which increased the overall acceptability of the platform.

Qualitative interview data collected in round 3 revealed features of gameplay and user experience that participants considered important to improve acceptability, including the use of sound feedback and motivating, familiar games. Participants suggested the addition of sound-based feedback to the game, indicating that they would enjoy hearing music (“Music would be great for them. They will train more if there is music” [P07, staff]) and sounds indicating their game performance (“What if you had sounds? One [sound] that is positive, a beep or a pause or you could do the other ones. Like in other words if it’s a failure or if you guess something wrong, it would go ‘buzzzz’. That would be it!” [P18, resident]). Audio feedback with simple tones was introduced in round 2, and we improved the audio to become more sophisticated in subsequent rounds, which was well received based on participant interviews. By round 4, the system was playing segments of familiar songs and could support better quality sounds with higher sample rates and featured user volume control. In total, 2 residents stated that they did “not like games in general” (P01 and P22) and, in later sessions, when both experienced playing a game on the exergame platform, they both appeared motivated and excited to engage with the technology as documented through observations. For instance, the residents wanted to continue playing and expressed excitement and disappointment at the last level. During the interviews, residents suggested games they wanted the team to develop and indicated that game selection would be a determining variable about whether they would use the MouvMat in their daily routines:

...it depends on the types of games that it provides. Like some games could be more mentally stimulating than others.

P22, resident

Residents suggested classic games such as snakes and ladders, scrabble, matching or memory games, bingo, tic-tac-toe, and checkers.

### Exergame Usability

The mean, SD, and range of SUS scores for each round are reported in Figure 1 and Multimedia Appendix 2. The mixed linear model predicting SUS scores showed a significant main effect of group (*F*_1,22_=13.46; *P*=.001), with higher usability ratings among staff and family members. SUS scores also significantly improved across the 4 rounds of testing (*F*_3,37.4_=14.30; *P*<.001). There was no interaction (*F*_3,37.4_=0.76; *P*=.52). Notably, the responses by the fourth iteration of the prototype exceeded the standard cutoff score for acceptable usability [42].

This is indicative of improved usability, as the team addressed the needs identified by residents and stakeholders in previous rounds, as summarized in Tables 2 and 3. A significant number of modifications were made to the hardware design of the MouvMat to increase usability, and changes were made based on observations and interviews while participants interacted with the prototype. Some examples of modifications based on observations included increasing the size of the squares to better accommodate larger shoe sizes, so they did not activate multiple squares at once; reducing the thickness of the squares and removing the wires to embed wireless connectivity to reduce fall risk and make it easier for residents with gait aids to navigate around the MouvMat; and changing the pitch and size of the LED lights to increase the definition of images while reducing glare, as some participants asked what shape or letter was on square. Observations of staff assembling and disassembling the MouvMat indicated some confusion matching the male-to-female physical connectors, and when we asked about that aspect, it was confirmed in the interview that staff preferred lighter squares with an automatic mechanism:

If I have to carry a bunch of these squares, it’ll be heavy to bring them up and down from floor to floor...I would like to just throw on the ground and have it assemble by itself without me thinking too much.

P07, staff

In response to this staff need, we redesigned the frame and replaced the male-to-female physical connectors with strong magnets that would self-assemble the MouvMat to make it as easy as possible for staff. In addition, we replaced the internal components to reduce the weight of the tiles. By observing how residents were holding the peripheral device and inquiring during the interview, it became clear that we needed to make changes to the peripheral accessory to make it lighter and more comfortable for the residents to hold and use. During the testing period, we designed and tested games that were based on residents’ interests as reported during semistructured interviews (refer to Multimedia Appendix 1 for game descriptions). To respond to stakeholder feedback, the games were programmed to be customizable at the speed and difficulty level to accommodate the user.

### Exergame Enjoyment

Figure 1 and Multimedia Appendix 2 outline the mean, SD, and ranges of PACES-8 scores from the 4 rounds. The mixed linear model predicting PA enjoyment scores from the PACES-8 revealed a significant main effect of group (*F*_1,32.7_=11.00; *P*=.002), with higher enjoyment ratings among staff and family members compared with residents. There was a significant increase in enjoyment scores for the MouvMat across the 4 usability sessions, as the games were modified (*F*_3,43.4_=19.47; *P*<.001). There was no interaction between the group and the testing session (*F*_3,43.4_=1.40; *P*=.25).

Importantly, semistructured interviews indicated that many participants thought that the MouvMat would be enjoyable to use, which would increase residents’ PA levels:

This encourages physical activity and I think that is useful. It was mentally stimulating and physically engaging, I quite enjoyed it.

P01, resident

All participants, including residents, agreed that increasing PA and cognitive stimulation would be beneficial. Specific characteristics of the system that were identified as important to enjoyment included the potential to individualize needs for independent or group use, a combination of cognitive stimulation with PA, increasing social interaction with others, and novelty of engaging in a new activity. Several staff participants shared that they felt the exergaming system could improve the autonomy of residents’ PA routines because it allows them the opportunity to engage in independent physical exercise:

Rather than having the physiotherapist go around to every single resident’s room, I think this is another way for the [residents] to be able to do [physical activity] on their own...either in their room or activity area.

P20, staff

In line with the research team’s goals, participants also spoke about how they felt that the system was an effective way to combine both cognitive and physical stimulation:

Yeah, I liked that it provided the cognitive and physical activity together. I like the idea because you are using both your mind and your body at the same time, so there should be coordination.

P24, resident

In addition, the opportunity to increase social interactions and engagement among residents in LTC during PA was perceived as a benefit of the MouvMat. Staff members shared during interviews that they believed MouvMat would increase residents’ current social circles by providing them with an opportunity to engage with other residents who they would not normally interact with, for example, residents could use the mat together or residents could watch others compete during gameplay. As explained by one staff member:

I think bringing two people together to play a game against each other or bring a group together to watch two people play against each other will build relationships and create social situations, interactions...they can cheer each other on.

P21, staff

Respondents liked that it provided a new opportunity for them to engage in a different task with others:

Yeah, I like movement and I like something new!

P03, resident

other than having it in their rooms, having [the MouvMat] in a public activity area could engage residents in games with other residents that they don’t usually talk to.

P21, staff

### Facilitators and Barriers to MouvMat Implementation

Through semistructured interviews with staff participants, we identified the facilitators and barriers to MouvMat implementation in the LTC setting. The 3 most frequently identified facilitators of new technology adoption in their LTC homes were the importance of empirical evidence to support the benefit of the technology to residents, ease of use, and versatility of the technology. Staff decision makers indicated that they take into account whether new interventions or technologies have any research or proven benefits for LTC residents. Staff also emphasized the need for the exergame design to be lightweight, compact, and quick to set up because of the heavy workloads. The life enhancement and activation therapists service the entire home, which requires them to travel between units and the various recreation rooms. In addition, the lack of physical space was identified as an issue, so new interventions need to be adaptable to fit different-sized common areas and activity rooms. Furthermore, because of the lack of space, exergames that are versatile and could provide a variety of activities that are customizable to the residents’ abilities are highly preferred. Technologies that considered the contextual realities of the LTC home that most residents could realistically use were more likely to be purchased.

The primary barrier to the acquisition of new technology in LTC home adoption is cost. All the staff stakeholders identified cost as a barrier and explained how available funds were influenced by different variables, including home fundraising efforts, the annual funding envelope to support activities, and whether the payment model for the technology was an outright cost or a subscription-based service requiring ongoing payments. The second barrier was a fear of harm or falls mentioned by 2 staff members; however, both respondents agreed that the accessory for wheelchair-bound residents would be an effective way to still have residents engage in upper limb activity while reducing fall risk.

## Discussion

### Principal Findings

This paper presents the development of a novel exergaming intervention co-designed with LTC residents with varying cognitive and physical abilities as well as the primary facilitators and barriers to the implementation of this exergaming platform in the LTC home setting. A robust UCD approach was used, which included validated measures and inputs from key stakeholders, such as LTC residents, their family members, and staff members. There were upward trends in the acceptability, usability, and enjoyment scores of MouvMat over time, indicating that the identified themes were appropriately addressed by the team. Key features that were identified by participants as important to increase exergame acceptability included the possibility of playing with it in either a seated or standing position, minimizing fall risk, intuitive gameplay requiring minimal instruction, auditory performance feedback, and familiar games. Improvements in usability across successive prototypes focused on the size, thickness, and weight of the squares, ease and comfort of using the peripheral accessory, and improving visual clarity of the display. Contributors to the enjoyment of the exergame included the opportunity for social interaction with other residents as well as the sense of novelty that the exergame provided. In addition, staff and stakeholders were drawn to MouvMat’s ability to increase residents’ autonomous PA. These findings are consistent with the current literature on habitual PA, which suggests that reducing health risk factors and maintaining functional abilities are among the most common self-reported intrinsic motivators for engaging in exercise among OAs [54-56].

OAs with cognitive impairment, as indicated by the MMSE score (Table 1), can also be included in exergame activities that use simple and familiar games. By actively engaging OAs residing in LTC homes and other stakeholders in the design process, researchers and developers can design more suitable exergaming interventions that avoid the limitations of existing commercially available exergames [19]. Our observations and interviews revealed that residents enjoyed engaging with the prototypes, appreciated the opportunity to increase their PA, and appreciated the novelty of something different from their usual routine in LTC. Besides the residents, staff were excited to see the development between the rounds and were very interested in the creation of the MouvMat. Our usability study demonstrates that an exergame can be co-designed to meet the diverse requirements (ie, hardware design features and motivation) and constraints (ie, residents’ physical and cognitive impairments) of end users. LTC residents are a historically challenging group to conduct research with [57-59], especially those with cognitive impairment [60-62], and as such have often been excluded from research [63]. An in-depth understanding of the complex physical, cognitive, and medical complexities of this population is imperative because LTC residents often experience symptoms, including fatigue and hearing or visual impairments, which can severely impact all aspects of the research, especially recruitment, retention, and data collection [58]. When collaborating with this population, it was important that all members of the team were knowledgeable about OAs in LTC; for instance, the 2 authors (CC and RB) are clinicians specializing in geriatrics, and the design team (led by HM) had previously created technologies for OAs. Special considerations when working with this population [62,64] included assessing assent, using effective communication strategies to fill out the validated tools, and conducting interviews. Researchers who have a strong understanding of the illness trajectory and who have experience with OAs can use appropriate strategies and approaches to ensure ethical and accurate data collection. Furthermore, additional considerations that may be unique to our study were that the setting was distraction free and private for testing and interviews, eliminating all safety risks during testing, such as checking for nonslip shoes and confirming that no recent medications were taken, as well as adjusting for the clinical realities of the LTC setting. Consistent with other studies [62], we also experienced user testing sessions being postponed or canceled if the resident was ill or had a medical appointment and scheduled for other activities, and residents often felt too lethargic to engage in PA after lunch; therefore, patience and open and empathetic attitudes from all team members were essential.

The development and adoption of innovative exergaming technologies for clinical settings, such as LTC, is inherently complex and is a context in which researchers must work within existing financial constraints. The main barriers to the implementation found in this study were the operational cost, followed by the risk of harm. The barriers to uptake in this study are corroborated by existing research in gerotechnology [65-67]. The most common facilitators reported were the intended and perceived benefits of MouvMat use, ease of use, and versatility of the technology, so that it can be used by a diverse group. MouvMat’s design minimizes fall risk and can be used from standing and seated positions and addresses accessibility issues faced by OAs with physical disabilities. Identifying facilitators and barriers is a foundational building block for generating a viable knowledge translation plan. Moving forward, we anticipate adopting an integrated knowledge translation approach [68,69], which is a continuation of the ethos of the UCD approach used in this study.

This project aims to create new exergaming technologies that are of value to the LTC sector and, in doing so, generate new scientific directions for the development and assessment of new exergaming platforms that can promote physical, cognitive, and social activity for LTC residents. In this context, it is important to develop appropriate exergaming interventions that meet the needs and interests of residents. Future research directions include conducting a pilot multisite randomized controlled trial to evaluate whether MouvMat can improve physical, cognitive, and psychosocial outcomes in LTC residents. Additional work with stakeholders to elucidate further facilitators and barriers to exergaming technology acquisition into LTC homes using an integrated knowledge translation approach would also be valuable. This research project has the potential to radically broaden our conceptualization of how to co-design exergaming technologies for OAs living in LTC homes.

### Strengths and Limitations

This study had several strengths. First, a user-centered approach was used and included residents with various levels of functional ability and cognitive function and other stakeholders, including staff members with different positions (eg, physiotherapist, life enhancement, and personal support worker) and family members. Second, we collected interview data and validated quantitative scales to measure participant acceptability, usability, and enjoyment to generate a comprehensive understanding of refinement needs. Third, the trustworthiness of the data was enhanced by member checking and triangulation across data sources (eg, observations, think-aloud responses, and interviews). Fourth, an independent observer was present on each testing day, which provided another perspective. Finally, an interdisciplinary team that included health care, psychologists, game developers, and engineers provided different perspectives.

The findings of this study should be interpreted with caution because of the following limitations. First, the study sample included a relatively small number of participants and included 2 mid- to large-sized nursing homes in Canada, which may impact the generalizability of the results to other contexts. Although a concerted effort was put forth by the team to get residents and stakeholders to complete all 4 rounds of usability testing, this was not feasible based on scheduling conflicts with appointments and illness or injury unrelated to the study. We also noted that difficulty retaining participants is a common challenge in research on OAs in LTC [62]. Nonetheless, most participants completed at least two rounds of user testing and were able to provide feedback, as the prototype became more sophisticated over time. It should also be noted that the analysis of the average MMSE scores of participants who completed the rounds indicated that selective attrition of participants with poorer cognitive function and frailty was not an issue; in particular, MMSE scores were lowest in participants who completed 3 rounds of testing. Second, the interviewer for some of the interviews was a member of the research team, which may have prevented participants from sharing negative opinions that criticized the exergame. We believe that the use of a question guide with open-ended questions and allowing the participants to speak freely, as well as triangulation [50,70] of the data, contributed to the validity of the results.

### Conclusions

Our study demonstrated that an exergaming platform could be cocreated with LTC home residents with multiple cognitive and physical impairments, who are a challenging group to engage in research. A user-centered, iterative design process was applied to successfully refine an exergaming platform to increase its acceptability, usability, and enjoyment for LTC residents. Facilitators and barriers to the future implementation of the MouvMat into LTC were identified, and this knowledge will contribute to an integrated knowledge translation plan. Cost was the major barrier to the acquisition of new technology; however, despite this barrier, residents and stakeholders were positive overall about its potential to improve OAs’ physical health and social engagement. The next research steps include determining the effectiveness of the MouvMat in a future multisite randomized controlled trial and developing analytics of game performance to indicate improvements or declines in physical and cognitive function. This research project focuses on safe and ethical cocreation of exergaming interventions with residents in LTC to address the exclusionary technology development process that traditionally overlooks LTC residents.

## Appendix

Multimedia Appendix 1 Descriptions and instructions of the games that were tested during the usability study.

Multimedia Appendix 2 Exergame usability, enjoyment and treatment evaluation inventory scores stratified by participant type and round of testing.

## Abbreviations

AGE-WELL NCE: Aging Gracefully Across Environments Using Technology to Support Wellness, Engagement and Long Life Networks of Centers Excellence
FRAIL-NH: Frailty in Nursing Homes Scale
LTC: long-term care
MMSE: Mini-Mental Status Examination
m-TEI: Modified Treatment Evaluation Inventory
OA: older adult
PA: physical activity
PACES-8: 8-item Physical Activity Enjoyment Scale
SDM: substitute decision maker
SUS: System Usability Scale
SWOT: strengths, weaknesses, opportunities, and threats
UCD: user-centered design
