# Supplementary material for: Exergaming Platform for Older Adults Residing in Long-Term Care Homes: User-Centered Design, Development, and Usability Study
Source: JMIR Serious Games. 2021 Mar 9;9(1):e22370. doi: 10.2196/22370 (PMC7988392; doi:10.2196/22370)
Supplement: Multimedia Appendix 1 [file games_v9i1e22370_app1.docx]

**Appendix A – Game descriptions and instructions of the games tested during the usability study**

| **Game** | **Description** |
| --- | --- |
| Bingo | “This is the game of Bingo. When you hear a letter, please go step/tap on that letter to get a star. The first player who creates a row of stars wins”  Using audio and visual, participants were asked to step or tap on the square that had the letter that was called out using audio stimulation. Each player would get their own 9 gaming squares. E.g. If “A” was called out on the audio the participant would get it correct if they stepped on “A” on the tile. |
| Don’t Let the Music Stop | “This game is called “Don’t let the music stop”. You will hear a part of a famous song. Then the squares will blink in a random order. You must step on the squares in the same order to hear the next part of the song”  A 10-second music clip of a popular song is played (e.g. Frank Sinatra, Elvis) and the player is shown a sequence of lights on the squares. He player must copy the sequence on the squares to activate the next clip of the song. |
| Matching Game | “This is a matching game. You will be shown images on the squares for 1 minute. The images will disappear. You must step on the squares you think match”  The participant is shown a series of pictures, one on each gaming square, and the images would disappear. The participant must step or use the peripheral accessory to tap on a square in order to match the images. If correct, the user would “match” a pair of images. If incorrect both squares would turn off and the participant would try again. |
| Memory pairs | “This is the a memory game. Step on the squares to reveal the image. Try to remember which squares show the same image and step on the tiles you think match.”  A classic game in which all of the gaming squares display the same image on the surface. The player must step or tap on two squares to reveal images, and match the cards. The object of the game is to turn over pairs of matching cards. |
| Scrabble | “This is the like the game of Scrabble. You will be shown a group of letters. Step on the letters to spell a word, then step on the tile that says Start”.  The squares would display alphabetical letters from the English language on five squares while the sixth square was a “submit/start” button. The participant would be asked to spell words using the letters that are visible by tapping and/or stepping on the tile. Once an entire word is spelled, they would tap or step on the “submit/start” tile. After tapping this, the system would register the word that the participant spelled. If correct, the participant would receive positive feedback from the system. |
| Simon Says | “This is the game of Simon Says. The squares will blink in a random order. You must step on the squares in the same order. Each light sequence will get progressively longer”  The classis game of Simon where a participant is shown a light sequence on the gaming squares and they must replicate the exact pattern by tapping and/or stepping on the tiles. The sequence of lights gradually gets longer up to a maximum number of 8 squares lighting up. The speed of this game is customizable. |
| Tic-tac-toe | “This is the game of Tic-tac-toe. Step on the square where you want to place your X. The person who get 3 X’s in a row is the winner.”  The game is played against the computer. Either the user starts by stepping on a square or the computer takes the initiative. The first player plays with a circle mark and the second with an “X” mark. To win, the player needs to have three of their marks in a horizontal, vertical, or diagonal row |
