# Supplementary material for: Exergaming Platform for Older Adults Residing in Long-Term Care Homes: User-Centered Design, Development, and Usability Study
Source: JMIR Serious Games. 2021 Mar 9;9(1):e22370. doi: 10.2196/22370 (PMC7988392; doi:10.2196/22370)
Supplement: Multimedia Appendix 2 [file games_v9i1e22370_app2.docx]

**Appendix B**

|  | **Round 1** | | **Round 2** | | **Round 3** | | **Round 4** | |
| --- | --- | --- | --- | --- | --- | --- | --- | --- |
|  | **Resident**  (n= 7) | **Staff/Family**  (n= 5) | **Resident**  (n=2) | **Staff/Family**  (n= 6) | **Resident**  (n= 6) | **Staff/Family**  (n= 6) | **Resident**  (n=9) | **Staff/Family**  (n=5) |
| **SUS Score (out of 100)** |  |  |  |  |  |  |  |  |
| Mean (SD) | 53.85 (11.90) | 68.33 (9.54) | 55.63 (14.38) | 69.58 (12.02) | 59.67 (7.26) | 76.67 (16.43) | 80 (10.34) | 85 (4.41) |
| Range | 37.5-70 | 50-82.5 | 70-77.5 | 47.5 – 77.5 | 52.5-75 | 52.5-97.5 | 55-90 | 77.5-90 |
| **PACES-8 Score (out of 56)** |  |  |  |  |  |  |  |  |
| Mean (SD) | 27.0 (8.91) | 38.5 (3.73) | 40.5 (0.5) | 42.67 (4.78) | 39.6 (9.89) | 49.17 (6.49) | 46.89 (6.81) | 50.89 (4.33) |
| Range | 13-40 | 32-43 | 40-41 | 40-49 | 25-56 | 37-56 | 41-56 | 47-56 |
| **m-TEI (out of 91)** |  |  |  |  |  |  |  |  |
| Mean (SD) | 62.83 (16.96) | 71.17 (5.21) | 65.5 (13.5) | 70.87 (4.56) | 63.7 (12.24) | 71.4 (9.05) | 71.6 (7.93) | 75 (5.79) |
| Range | 35-85 | 61-77 | 52-79 | 64-79 | 46-76 | 58-79 | 52-79 | 69-87 |

SUS: Systems Usability Scale, PACES-8: Physical Activity Enjoyment Scale; m-TEI: Modified Treatment Evaluation Inventory
